# Networking in the Plant Microbiome

**DOI:** 10.1371/journal.pbio.1002378

**Published:** 2016-02-12

**Authors:** Marcel G. A. van der Heijden, Martin Hartmann

**Affiliations:** 1 Plant-Soil-Interactions, Agroscope Institute for Sustainability Sciences, Zurich, Switzerland; 2 Department of Evolutionary Biology and Environmental Studies, University of Zurich, Zurich, Switzerland; 3 Plant-Microbe Interactions, Institute of Environmental Biology, Faculty of Science, Utrecht University, Utrecht, The Netherlands; 4 Forest Soils and Biogeochemistry, Swiss Federal Institute for Forest, Snow and Landscape Research, Birmensdorf, Switzerland

## Abstract

Almost all higher organisms, including plants, insects, and mammals, are colonized by complex microbial communities and harbor a microbiome. Emerging studies with plants reveal that these microbiomes are structured and form complex, interconnected microbial networks. Within these networks, different taxa have different roles, and keystone species have been identified that could be crucial for plant health and ecosystem functioning. A new paper in this issue of *PLOS Biology* by Agler et al. highlights the presence of microbial hubs in these networks that may act as mediators between the plant and its microbiome. A next major frontier is now to link microbiome composition to function. In order to do this, we present a number of hypothetical examples of how microbiome diversity and function potentially influence host performance.

## Abstract 2

This Primer assesses recent evidence that the microbiome of plants contains microbial networks and keystone species that influence plant health and ecosystem functioning. Read the accompanying Research Article.

## The Microbiome

Microbial communities play a central role in virtually every biogeochemical cycle on earth, driving global carbon and nutrient cycling with direct feedback on ecosystem functioning and productivity. A multitude of microorganisms also associate with higher organisms and collectively function as a microbiome. It is now well established that every higher organism investigated, from plants, insects, and fish up to mice, apes, and humans, harbors a microbiome (Table 1). Findings in the last decade revealed that these microbial communities do not simply inhabit our skin or intestine but also appear to influence processes including behavior, appetite, and health [1,2].

**Table 1:** Selection of host taxa for which the microbiome has been assessed.

Mammals*	Other Animals*	Plants*
Human	Zebrafish	Barley
Elephant	*Hyrda*	Maize
Chimpanzee, gorilla	Termites	*Arabidopsis* (Thale cress)
Rabbit	Cockroaches	Grapevine
Polar Bear	Springtails	Rice
Horse	Thrips	Hairy bittercress

*Adapted from [3–5]. Note that many studies investigating microbiomes focused on specific taxonomic groups (e.g., prokaryotes), and other important domains (e.g., fungi or protozoa) have often not been assessed.

While research on the human microbiome is booming, less attention has been paid to the microbiome of plants. It has long been recognized that plants associate with microbes, and a wide range of studies have demonstrated that plant organs, such as roots or leaves, harbor complex and diverse communities of fungi and bacteria that live either inside the tissue or on the surface of these organs [4,6,7]. Up to a few thousand bacterial and fungal taxa colonize plant roots and leaves [4,6,7], and sometimes bacterial communities on leaves and roots can be surprisingly similar [8]. Recent studies, including the work by Agler et al. [9] in this issue of *PLOS Biology*, explored the microbiome of plant leaves, specifically focusing on the effects of host genotype, the abiotic environment, and the role of specific microbial taxa in determining microbiome composition and interactions among various microbial taxa.

Agler et al. [9] used high-throughput sequencing to characterize the microbiome of *Arabidopsis thaliana*, the model plant for molecular biology and genetics. They sampled the microbiome of *Arabidopsis* leaves collected in the wild, and they performed experiments in the greenhouse. While earlier studies mainly targeted prokaryotic members of the microbiome, the study by Agler et al. also included fungi and oomycetes. The field experiments revealed that both abiotic factors and plant genotype influenced microbiome composition, and they observed that specific microbes (e.g., the obligate plant pathogen *Albugo*, the fungus *Dioszegia*, and a few others) significantly influenced microbiome structure. In a following set of greenhouse experiments, Agler et al. experimentally manipulated the microbiome of different plant genotypes by adding *Albugo* and *Dioszegia*, and they could prove that these microbes act as keystone species and are major determinants of the microbiome network structure. The abundance of the keystone species *Albugo* depended very much on whether or not a plant genotype was resistant to this plant pathogen. In addition, and perhaps most interestingly, the findings from Agler et al. suggest that the plant host genotype acts on keystone microbial species, which then transmit these effects upon the whole microbial community by modulating microbe–microbe interactions and changing host fitness.

## Microbiome Networks

The functional capacity of the plant microbiome is not equal to the sum of its individual components, since microbial species strongly and frequently interact with each other and form a complex network. A range of tools have been developed to analyze such networks, and these are widely used by biologists, mathematicians, social scientists, and computer scientists to explore interaction between entities, whether those individuals are students in a school, species in a food web, nodes on a computer network, or proteins in metabolic pathways [10,11]. Microbial networks often consist of thousands of interdependent constituents that interact in mutualistic, synergistic, commensalistic, ammensalistic, or parasitic modes. These interactions have the potential to influence each constituent’s fitness, with direct consequences for soil fertility and plant health. Understanding these microbe–microbe interactions is crucial in order to predict the holistic consequences of these interactions for plant physiology and performance.

**Fig 1:**
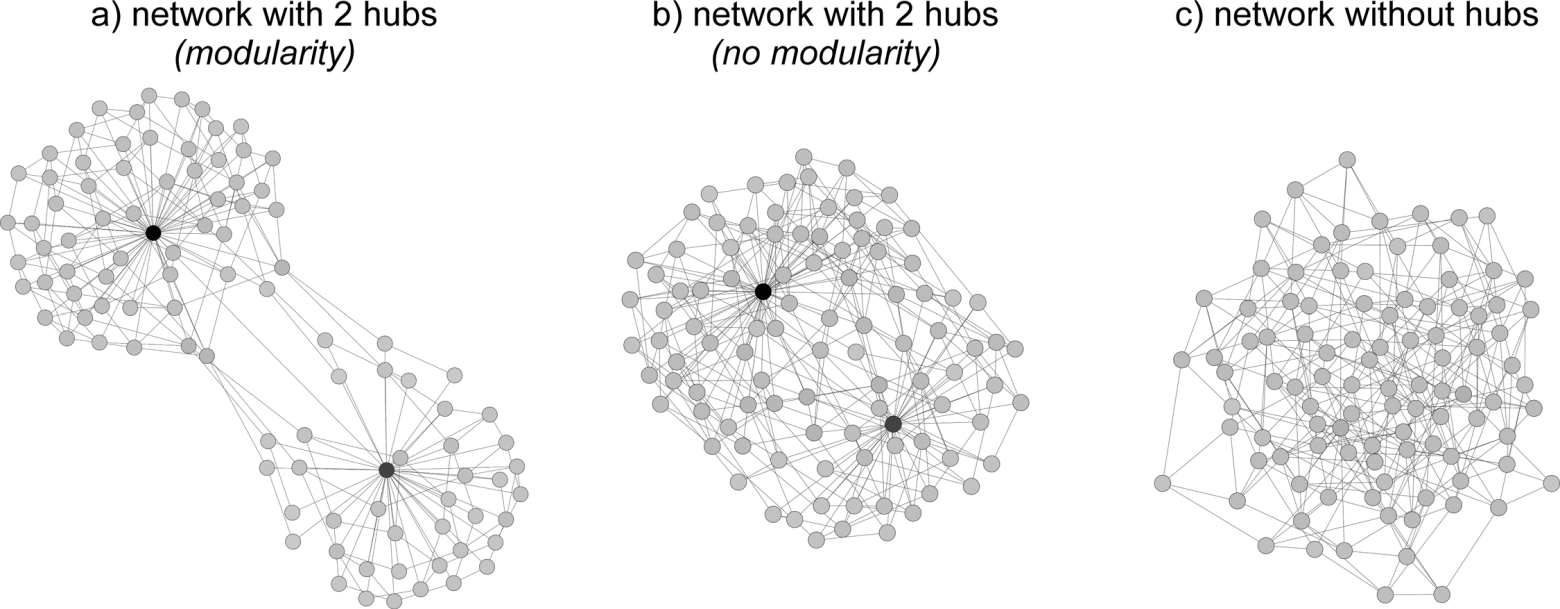
Microbial networks vary in complexity and organization. (A) A compartmentalized microbial network consisting of two hubs of microbial taxa that frequently interact and/or respond similarly to environmental cues. (B) A microbial network with two hubs but without modularity, as compartmentalization does not necessarily have to be observed in networks with hubs. (C) A strongly connected microbial network without hubs, where all taxa show similar degrees of interactions.

A useful approach to gain a better understanding of potential interactions within the microbial network is to construct co-occurrence networks. These co-occurrence networks are usually established by calculating correlations between the abundance of individual entities (e.g., microbial taxa). Microbial taxa that frequently co-occur with other taxa in microbial co-occurrence networks potentially play a key role within the microbiome, as they might interact with many other microbial taxa. Such keystone species potentially have a large regulatory effect on their environment and other members of the microbiome (Fig 1). In contrast, microbial taxa whose abundance does not correlate with other microbes, which we call peripheral species, are most likely unaffected by other microbes in the network and have lower rates of microbe–microbe interactions. A key question for further research is whether microbiome functioning depends on organization, complexity, and interconnectivity of the microbial network.

Agler et al. [9] used the term “microbial hubs” for the presence of strongly interconnected species in the microbial network of plants. Their findings indicate that such highly interconnected species are important for plant health, as they mediate between the plant and the microbiome. In other words, the plant acts on the microbial hub, which then transmits the information to the broader microbial network, and likely vice versa. In this light, microbial hubs might recruit beneficial organisms or prevent invasion of pathogens in order to improve their own fitness, for the benefit of the whole system. Alternatively, if some of the hub species act as pathogens, as in the study by Agler et al., such species might initiate colonization by microbes that otherwise would not colonize the plant leaf surface, resulting in an alternative state of the microbiome. It is important to consider that co-occurrence networks report on correlations, not necessarily interactions among taxa. It is possible that a microbial hub species (e.g., a pathogen or a mutualist) indirectly influences many other taxa by changing host quality or performance without directly interacting with other microbes.

The framework of microbial hubs gives rise to novel ideas for sustainable management of soils and crops, as the presence of microbial hubs, or the loss of microbial hubs under certain environmental perturbations, could be critical for soil fertility and plant health. Microbial hubs might be responsible for sustaining disease-suppressive soils, improving nutrient uptake or effectiveness of biocontrol agents, and mediating defense signals among plants. Alternatively, microbial hubs in plant roots might facilitate disease development in a similar way, as the microbiomes of obese mice or humans facilitate the development of obesity [2]. It has already been shown that keystone microbial species (e.g., mycorrhizal fungi) can function as plant-to-plant communication systems by transferring information on disease resistance from pathogen-infected to healthy neighboring plants [12], or that plants recruit certain key species to ward off pathogen attacks [13]. Understanding identity, ecology, and physiology of such keystone microbial species and their interactions within the microbial network could be crucial for both sustaining plant health and soil fertility and could provide a potential target for novel agricultural management strategies.

**Fig 2:**
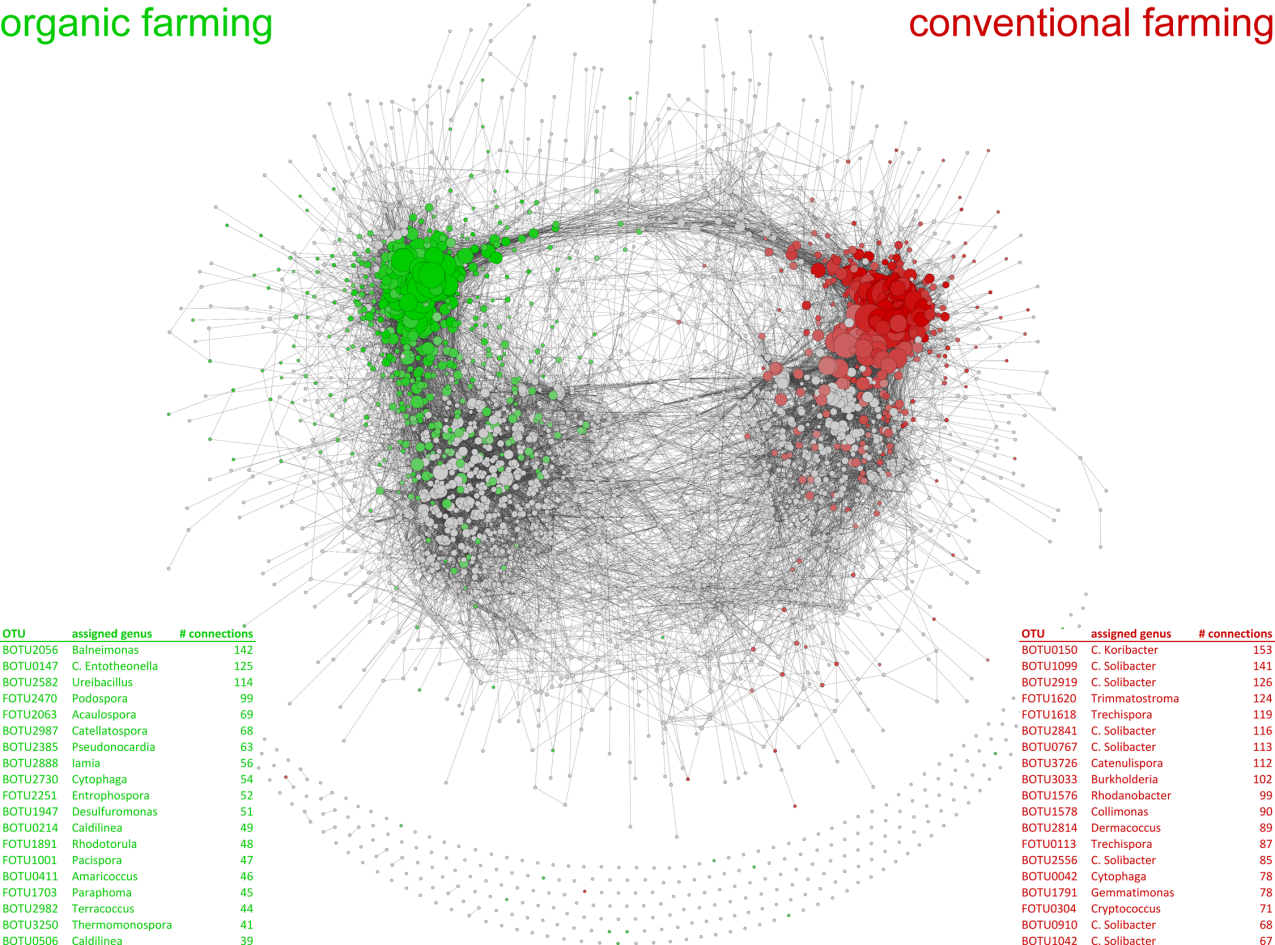
Co-occurrence network of microbial taxa detected in organically and conventionally managed soils by a high-throughput DNA sequencing approach of ribosomal markers (data modified after Hartmann et al., 2015 [14]). Nodes represent over 3,000 bacterial and fungal taxa, whereas edges represent significant positive correlations between pairs of taxa. Node size corresponds to the number of connections, and taxa with many correlations are located in the densely connected areas of the network. Green nodes are microbial taxa that are significantly more abundant in organically managed plots, while red nodes are significantly more abundant in conventionally managed plots. Taxa that showed highest connectivity in the two systems, and which could be assigned at genus level, are indicated as tables in the left (organic) and right (conventional) corner of the plot (see S1 Text for further details).

It was recently shown that microbial communities from conventional and organically managed fields vary considerably [14], even though these fields had the same history and share exactly the same soil type and climate. This demonstrates that different agricultural management practices select for different microbial species and communities [14]. Microbial co-occurrence networks of both agricultural management systems yield very different microbe–microbe connectivity patterns (Fig 2), suggesting that different microbial hubs could be important in the organic and conventional systems. Both farming systems feature several highly connected hubs, but of different identities (Fig 2). Several of the highly connected taxa in this co-occurrence network have been suggested to be plant-associated microorganisms, indicating that these taxa could indeed function as hubs within the plant microbiome. Interestingly, there is no correlation between taxon abundance and connectivity, also suggesting that low-abundance taxa can be highly interconnected and potentially function as microbial hubs, or that highly abundant taxa are poorly connected (S1 Fig).

## Plant Microbiome Performance

While the factors that drive plant microbiome composition are now better understood, a major challenge for future research is to link microbiome composition to function. For instance, is it possible to use and manipulate the plant microbiome to enhance agricultural productivity or environmental sustainability? The finding by Agler et al. [9] that plant genotype has a big impact on microbiome composition indicates that breeding and genotype selection offers opportunities to select for specific (beneficial) microbiomes. Moreover, the observation by Agler et al. that plant genotypes can influence the microbiome by acting on keystone species suggest that it is possible to influence the microbiome assembly process by selecting particular plant genotypes. This is an area that has only recently been explored, and many exciting discoveries likely wait in the future. In order to answer these questions, there is a need to integrate novel molecular technologies (e.g., metagenomics, metatranscriptomics), ecological theories (e.g., food web theory, neutral theory on the stochastic assembly of communities, or theory that explains coexistence of species by different niches), and recent computational and statistical advances (e.g., structural equation modeling) in order to link community composition and dynamics with ecological functions.

Many microbes simply inhabit the plant microbiome as colonizers of the phyllosphere (i.e., aboveground compartment of plants as a habitat for microorganisms) or the rhizosphere (i.e., soil in the close vicinity of the root system and directly influenced by root secretions and associated soil microorganisms), using plant exudates for their growth. Such symptomless colonizers usually have no major impact on plant growth or fitness. However, a wide range of other microbes, including arbuscular mycorrhizal fungi, nitrogen-fixing bacteria, root endophytes, and a range of disease-suppressive bacteria do impact plant growth or nutrient acquisition positively [15]. Conversely, plant pathogens can invade the microbial network and have a negative impact on plant growth or fitness if they overcome plant defense. It is now a major challenge to investigate how these different microbes interact and influence plant growth.

## Linking Composition and Function

Studies focusing on particular functional groups of microbes revealed that the relationship between microbial diversity in the rhizosphere and plant performance can be positive. For instance, Maheraldi and Klironomos (2007) [16] manipulated the composition and diversity of arbuscular mycorrhizal fungi, widespread soil fungi that assist plants with nutrient uptake. They observed that plant performance increased with increasing mycorrhizal fungal diversity. Moreover, a recent study showed that a tripartite plant–fungal–bacterial symbiosis in plant roots substantially enhanced plant biomass and survival [17]. This indicates that different functional groups of microbes can complement each other with positive effects on plant growth. Further studies need to investigate whether such positive interactions between different microbes within the microbiome are rare or widespread and important for many plant species. Furthermore, a study by Mendes et al. [18] showed that a microbial consortium inhabiting the rhizosphere can suppress disease. Interactions between various groups of bacteria (e.g., members of the Pseudomonadaceae, Burkholderiaceae, Xanthomonadales, and Actinobacteria) were thought to be responsible for disease suppressiveness in that study.

**Fig 3:**
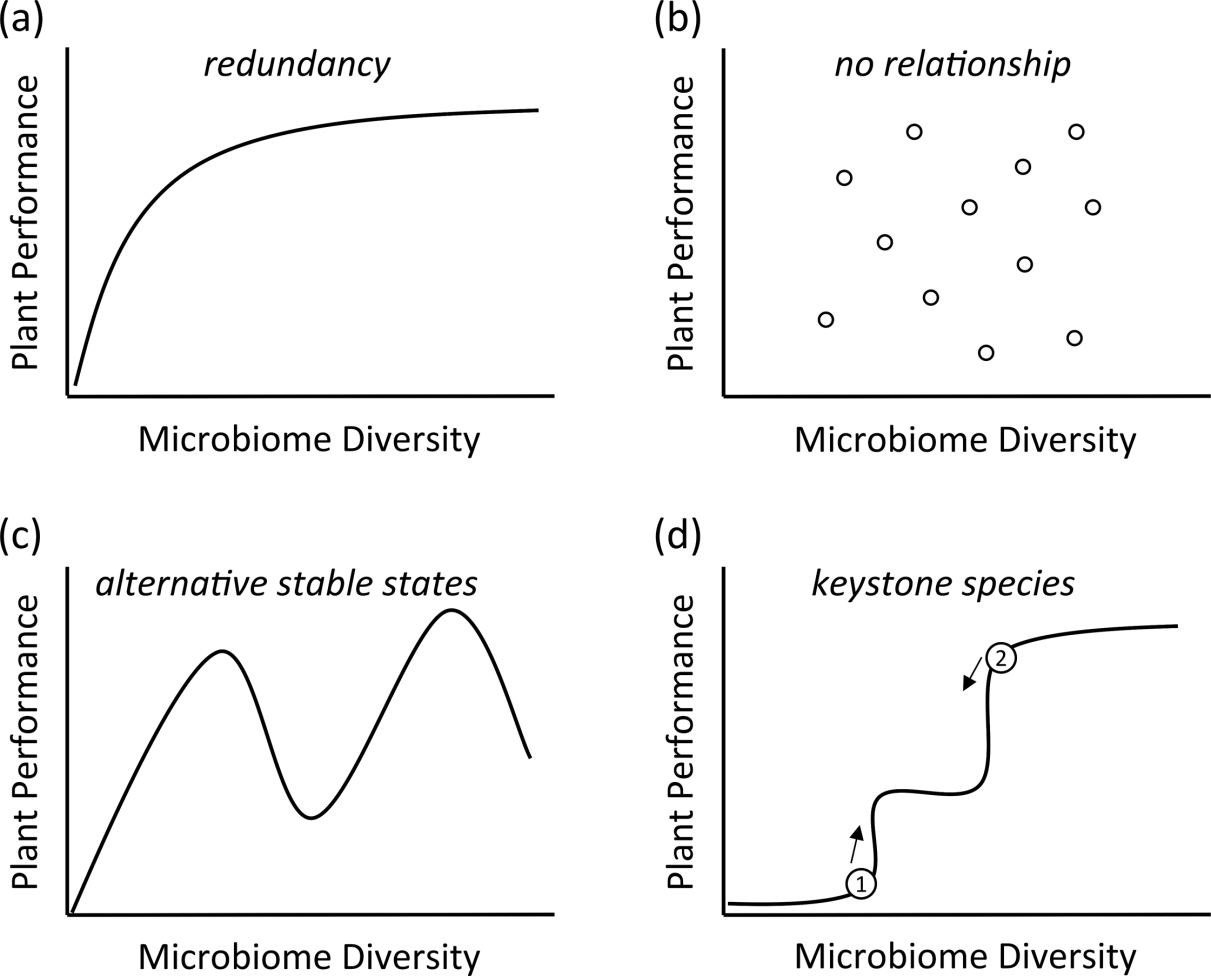
Hypothetical relationships between microbiome diversity and plant growth response. (A) The redundancy hypothesis predicts that plant performance increases with increasing microbiome diversity, but that saturation occurs in that further increases in diversity do not enhance performance, as additional microbial species are redundant. (B) There is no relationship between microbiome diversity and plant performance, and the plant response depends on factors such as plant species identity, the specific microbiome composition, and the biotic and abiotic environment. (C) There are alternative stable states and performance optima and minima depending on changes within the microbiome composition. For instance, the establishment of keystone species facilitates establishment of other microbes, leading to an alternative stable state. This, in turn, can alter plant performance. (D) The establishment (1) or loss (2) of a keystone species (numbered 1 or 2) has a big impact on plant responses, be it positive (as in this graph) or negative, when the presence of a pathogen reduces plant performance (modified after Naeem et al. 2002 [20]).

The major question is now whether it is possible to predict plant performance based on microbiome composition and diversity. Plant community ecologists have posed similar questions but at a different level of organization, namely at the plant community level. In the past two decades, a wide range of researchers have tested whether a link between plant diversity of a community and ecosystem functioning existed [19]. A number of relationships between plant diversity and ecosystem functioning have been found or are postulated [19,20]. Similarly, a number of hypothetical relationships between microbiome composition and diversity and plant performance can be proposed (Fig 3).

One hypothesis is that plant performance increases with increasing microbiome diversity and that, at a certain level of diversity, further increases in microbiome diversity do not lead to better plant performance (Fig 3A). The fact that many plant species cannot survive in absence of microbes [15], or that the diversity of arbuscular mycorrhizal fungi in plant roots enhances plant productivity, suggests that a positive relationship between diversity and performance exists, especially at the lower end of the relationship, where microbial diversity is very low. Moreover, it is also possible that plant microbiome diversity is unrelated to plant performance (Fig 3B), being dependent instead on particular hosts in combination with microbial taxa.

A further hypothesis is that plant performance depends on the presence of particular microbial species (e.g., keystone species), and that alternative stable states with high or low plant performance can be identified, depending on microbial community composition (Fig 3C). In ecosystems, alternative stable states and catastrophic shifts have been described that are linked to major changes in community composition [21]. Similarly, the arrival and establishment of a microbial pathogen in a microbiome, as shown by Agler et al., can have a dramatic effect on microbiome composition and plant performance. Related to this, it is important to investigate whether the composition of plant microbiomes reaches a stable, steady state [22] or whether the plant microbiome is a dynamic entity and changes with root or leaf age. A further hypothesis is that there are certain keystone taxa, and that the arrival of such taxa has a strong impact on microbiome assembly, and this, in turn, can have a positive (e.g., when mutualists arrive) or negative (e.g., when colonization through a plant pathogen occurs) impact on performance (Fig 3D).

The molecular tools to rapidly characterize the plant microbiome are now available, as shown by Agler et al. and other studies [4,6–9,14,18,22], and the time is ripe to move on to the next challenge and link microbiome composition with function.

## Supporting Information

S1 Fig **(A)** Co-occurrence network of microbial taxa detected in organically and conventionally managed soils. The taxa in this figure are scaled based on their relative abundance, and node size corresponds to the relative abundance of each taxa. This figure contrasts with Fig 2 in the main text, in which the taxa are scaled according to connectivity and node size corresponds to the number of connections. The numbers within each node refer to the number of connections of that particular microbial taxa. Thus, this figure shows that some taxa can be highly abundant (as shown by their node size) but less well-connected with other taxa (as shown by the number of connections within the node). Green nodes are microbial taxa that are significantly more abundant in organically managed plots, while red nodes are significantly more abundant in conventionally managed plots. Microbial communities were analyzed in four replicated plots for each farming system and across two different years. The farming systems differed in fertilization (organic versus mineral fertilization) and plant protection strategies (mechanic versus chemical pest control), whereas other parameters such as tillage and crop rotation were the same (see Hartman et al. [14] for specific details on the conventional [CONMIN] and organic [BIODYN] treatments of the DOK trial). **(B)** There was no significant relationship between abundance (number of sequences) and connectivity (number of significant positive Spearman correlations with r ≥ 0.6 and *p* < 0.001) for all taxa in the organically and conventionally managed soils. The correlation coefficient (R^2^) and the level of significance are provided in the right corner.
(TIF)

S1 Text Extended legend for Fig 2. (DOCX)
