# Supplementary figures and images for: Networking in the Plant Microbiome
Source: PLoS Biol. 2016 Feb 12;14(2):e1002378. doi: 10.1371/journal.pbio.1002378 (PMC4752285; doi:10.1371/journal.pbio.1002378)

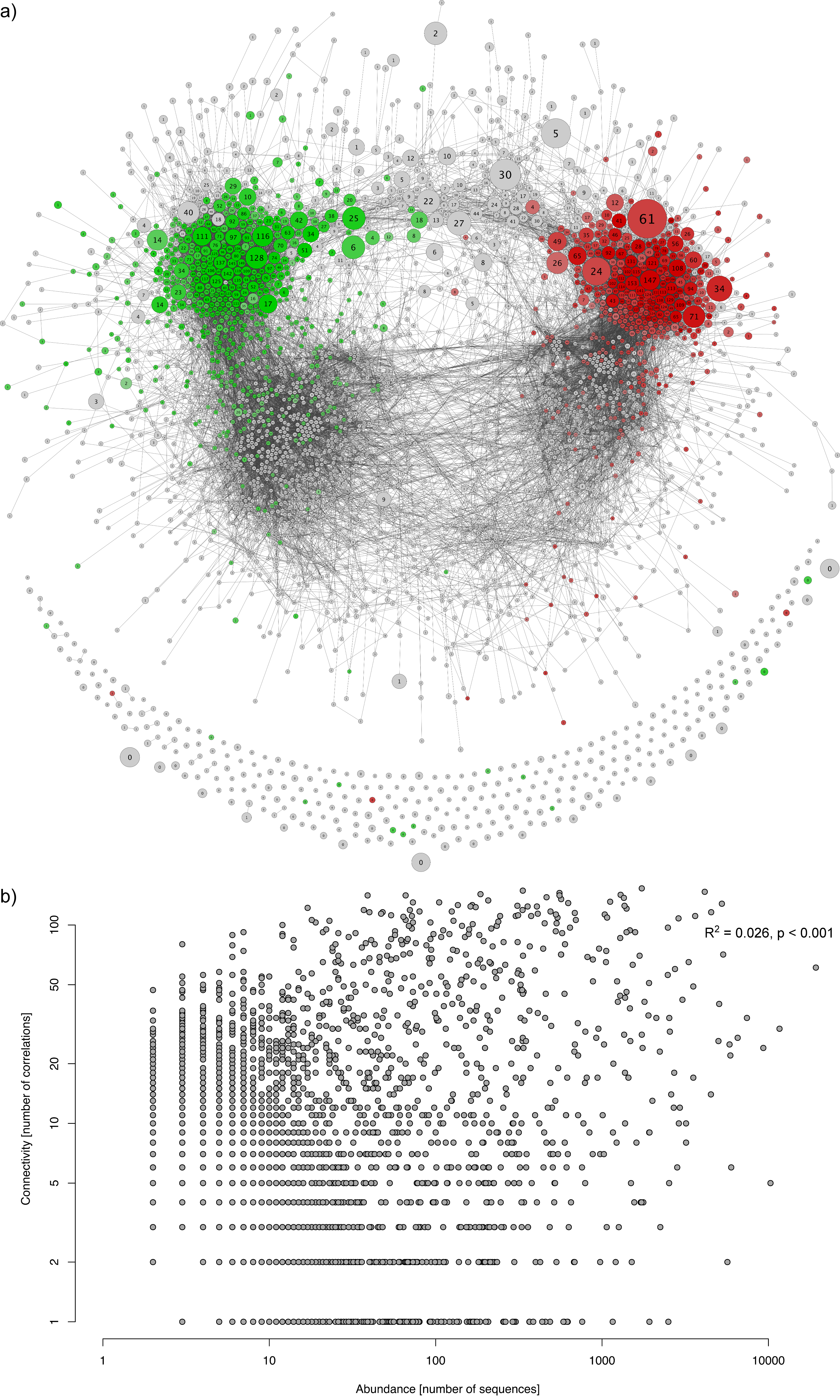

Supplement: S1 Fig — (A) Co-occurrence network of microbial taxa detected in organically and conventionally managed soils. The taxa in this figure are scaled based on their relative abundance, and node size corresponds to the relative abundance of each taxa. This figure contrasts with Fig 2 in the main text, in which the taxa are scaled according to connectivity and node size corresponds to the number of connections. The numbers within each node refer to the number of connections of that particular microbial taxa. Thus, this figure shows that some taxa can be highly abundant (as shown by their node size) but less well-connected with other taxa (as shown by the number of connections within the node). Green nodes are microbial taxa that are significantly more abundant in organically managed plots, while red nodes are significantly more abundant in conventionally managed plots. Microbial communities were analyzed in four replicated plots for each farming system and across two different years. The farming systems differed in fertilization (organic versus mineral fertilization) and plant protection strategies (mechanic versus chemical pest control), whereas other parameters such as tillage and crop rotation were the same (see Hartman et al. [14] for specific details on the conventional [CONMIN] and organic [BIODYN] treatments of the DOK trial). (B) There was no significant relationship between abundance (number of sequences) and connectivity (number of significant positive Spearman correlations with r ≥ 0.6 and p < 0.001) for all taxa in the organically and conventionally managed soils. The correlation coefficient (R2) and the level of significance are provided in the right corner. (TIF) [file pbio.1002378.s001.tif]
