# Supplementary material for: Networking in the Plant Microbiome
Source: PLoS Biol. 2016 Feb 12;14(2):e1002378. doi: 10.1371/journal.pbio.1002378 (PMC4752285; doi:10.1371/journal.pbio.1002378)
Supplement: S1 Text — (DOCX) [file pbio.1002378.s002.docx]

**S1 Text: Extended legend for Fig 2**

Co-occurrence network of microbial taxa detected in organically and conventionally managed soils by a high-throughput DNA sequencing approach of ribosomal markers (data modified after Hartmann et al. 2015 [14]). Nodes represent over 3000 bacterial and fungal taxa, whereas edges represent significant positive Spearman correlations (r≥0.6, p<0.001) between pairs of taxa. The edge-weighted Allegro Fruchterman-Reingold algorithm clusters taxa that strongly co-occur. Therefore, taxa with many correlations are located in the densely connected areas of the network; less connected entities exist in the peripheral part of the network. The taxa in this network are scaled based on connectivity and node size corresponds to the number of connections. Green nodes are microbial taxa that are significantly more abundant in organically managed plots while red nodes are significantly more abundant in conventionally managed plots. Taxa that showed highest connectivity in the two systems, and which could be assigned at genus level, are indicated as tables in the left (organic) and right (conventional) corner of the plot. Highly interconnected species in the conventionally managed system included several members of the acidobacterial candidate genera *Solibacter* and *Koribacter*. The organic system featured different highly interconnected taxa including the Proteobacteria *Balneimonas* (new *Microvirga*, a potential root-nodule bacteria), *Candidatus Entotheonella* , the Firmicutes *Ureibacillus* (a genus often found in manure and compost), the Actinobacteria *Catellatospora* (a potentially plant-associated genus) , as well as the fungi *Podospora* (coprophilic fungi on manure) and *Acaulospora* (an arbuscular mycorrhizal fungus). Approximately 10% of all microbial taxa in the co-occurrence network of organic and conventionally managed soils are poorly connected (see unconnected taxa at the bottom of Fig 2). The Microbial communities were analyzed in four replicated plots for each farming system and across two different years. The farming systems differed in fertilization (organic versus mineral fertilization) and plant protection strategies (mechanic versus chemical pest control), whereas other parameters such as tillage and crop rotation were the same (see Hartman et al. 2015 [14] for specific details on the conventional (CONMIN) and organic (BIODYN) treatments of the DOK trial). Further information (e.g. the number of connections per taxa) and the scaling of this co-occurrence networks according to taxon abundance is given in (S1 Fig).
